# Heat Scanning for the Fabrication of Conductive Fibers

**DOI:** 10.3390/polym13091405

**Published:** 2021-04-26

**Authors:** Jina Jang, Haoyu Zhou, Jungbae Lee, Hakgae Kim, Jung Bin In

**Affiliations:** 1 Soft Energy Systems and Laser Applications Laboratory, School of Mechanical Engineering, Chung-Ang University, Seoul 06974, Korea; jangjina94@cau.ac.kr (J.J.); skzzjd@cau.ac.kr (J.L.); haggai514@cau.ac.kr (H.K.); 2 Department of Intelligent Energy and Industry, Chung-Ang University, Seoul 06974, Korea; zhouhaoyu@cau.ac.kr

**Keywords:** sintering, conductive fiber, silver nanowire, silver nanoparticle, force sensor

## Abstract

Conductive fibers are essential building blocks for implementing various functionalities in a textile platform that is highly conformable to mechanical deformation. In this study, two major techniques were developed to fabricate silver-deposited conductive fibers. First, a droplet-coating method was adopted to coat a nylon fiber with silver nanoparticles (AgNPs) and silver nanowires (AgNWs). While conventional dip coating uses a large ink pool and thus wastes coating materials, droplet-coating uses minimal quantities of silver ink by translating a small ink droplet along the nylon fiber. Secondly, the silver-deposited fiber was annealed by similarly translating a tubular heater along the fiber to induce sintering of the AgNPs and AgNWs. This heat-scanning motion avoids excessive heating and subsequent thermal damage to the nylon fiber. The effects of heat-scanning time and heater power on the fiber conductance were systematically investigated. A conductive fiber with a resistance as low as ~2.8 Ω/cm (0.25 Ω/sq) can be produced. Finally, it was demonstrated that the conductive fibers can be applied in force sensors and flexible interconnectors.

## 1. Introduction

Recently, conductive fibers have attracted great attention for various potential applications such as electromagnetic interference shielding [1], fiber-type sensors [2,3], energy storage devices [4,5], and other multifunctional devices that require a highly flexible filamentous structure for electrical signals or power transmission [6]. The convergence of textiles and electronics has yielded e-textiles, which feature the integration of electronic components and interconnections into fabrics [7]. In an e-textile, the conductive fiber plays a pivotal role as a one-dimensional building block. For the commercial use of conductive fibers, not only should the fabrication cost be affordable, but the fibers should also maintain high conductance and functionality under severe mechanical deformation. Especially for wearable devices, the overall performance of the devices often depends on flexibility and stability against external deformation.

For the fabrication of conductive fibers, conductive materials have been integrated with polymeric fibers because pure metallic wires are heavy and vulnerable to repeated large-strain deformation [8]. The uses of conductive polymers or carbon nanomaterials as conductive materials have been explored [9,10,11]. However, although such materials exhibit excellent flexibilities, which indicate stable electrical conduction under mechanical deformation, they have much lower conductivities than metals. Thus, various techniques have sought to fabricate high-performance conductive fibers by combining metals and polymeric fibers, which exploits the high conductivities of metals and excellent flexibility of polymers. For example, metal wires can be physically mixed with polymeric filaments by winding or spinning as yarn [12,13]. However, this method presents limitations akin to those of pure metal wires. Specifically, small metal particles or thin layers of metal can be precipitated in polymer fibers via vapor deposition [14], metal plating, or other chemical processes that involve the reduction and sorption of metal precursors [2,15,16,17,18]. Although the resultant fibers exhibit enhanced mechanical flexibilities, the fabrication method is complicated and requires the use of harmful chemicals.

The reasons mentioned above motivate the use of simple dip coating methods to deposit metallic nanomaterials in polymeric fibers. This involves dipping polymeric fibers into an ink-like solution that contains dispersed metal nanoparticles [19]. By this method, the contact resistance between deposited individual nanomaterials is high due to the small contact area and capping agents added for solubilization. Thus, sintering is an indispensable process to fully access the high conductivity of the metal. Various sintering techniques have been developed, most of which use heat to induce sintering [20,21,22,23,24,25]. Annealing metal-deposited fibers by using an oven heater should be the most straightforward approach for sintering. However, because the supporting polymers are unstable at the sintering temperatures required for metals (except for specialized heat-resistant polymers) [26,27], annealing with conventional heaters is not an option, especially for small-diameter polymer fibers. The use of metal nanomaterials of smaller sizes may lower the sintering temperature, but this can increase material costs. Low-temperature sintering techniques, which exploit local heating by lasers [22,23], intense pulsed light [24], and plasma [21,28], can sinter metal particles with minimal damage to polymers. However, the non-planar geometry of polymeric fibers limits the applicability of these methods.

In this study, droplet-coating and heat-scanning methods were developed to fabricate flexible and conductive metal-deposited polymer fibers. Silver nanoparticles (AgNPs) and silver nanowires (AgNWs) were used as conductive elements because of their high conductivity and chemical resistance to corrosion. The AgNP and AgNW composite films show excellent conductance and flexibility when implemented on a flexible substrate [29]. The surface of a nylon monofilament (a single fiber) was coated with silver (Ag) nanomaterials by using a Ag ink droplet, and the coated fiber was subsequently subjected to heat scanning to induce sintering of the deposited Ag nanomaterials. The heat-scanning method provided uniform heating along the periphery of the fiber with minimal thermal damage. Finally, the conductive fiber was used to fabricate fiber-type force sensors and flexible interconnectors, which can be incorporated into e-textiles.

## 2. Materials and Methods

### 2.1. Coating of Ag Nanoparticles and Ag Nanowires

Commercial nylon monofilament fibers (diameter: ~280 μm; Justron DPLS, Daiwa Co., Tokyo, Japan) were cleaned in a solution of isopropyl alcohol (IPA) and deionized (DI) water (1:1 in volume) by using an ultrasonic cleaner (UP-02, Jeio Tech, Inc., Seoul, Korea) for 1 min and dried in a convection oven (forced convection type, SH-DO-90FH, Samheung Co., Seoul, Korea) at 50 °C for 10 min. To promote the adhesion of AgNPs and AgNWs to the fiber surface, the nylon fiber was submersed in a solution of ethyl acetate (99.9%, HPLC, Fisher) and resorcinol (≥99.0%, ACS reagent, Sigma Aldrich, St. Louis, MO, USA) (weight ratio = 91:9) for 2 min and then dried in air [19]. The fiber was then coated with AgNPs and/or Ag NWs via the droplet-coating method developed in this study. For the coating, AgNP ink (10 wt.% of nanoparticles in IPA, avg. diameter: 50 nm, SNP-050c, SG Flexio Co., Daejeon, Korea) and AgNW ink (0.5 wt.% of nanowires in IPA, avg. diameter: 40 nm, avg. length: 20 μm, SNW-006b, Flexio Co.) were used. For droplet-coating, the fiber was horizontally suspended and translated through the droplet (vol: ~10 μL) by using a motorized linear stage at 2.5 mm/s. The linear stage passed the fiber back and forth eight times (16 sweeps in total). Finally, the coated nylon fiber was dried in a convection oven at 50 °C for ~1 h.

### 2.2. Heat-Scanning System

A coil-type Joule heating element was prepared by winding a nichrome wire (diameter: 0.5 mm) around a cylindrical rod 15 times. The nichrome coil was ultrasonically cleaned in a mixture of IPA and DI water (1:1 in volume) for 15 min and then in 1 M sulfuric acid (H_2_SO_4_) for 5 min. The wire was cleaned again in the same IPA/DI water mixture for 15 min. To prevent the nichrome wire from oxidizing in air at high temperatures, a mixture of alumina cement (Insultemp cement no. 10, Sauereisen, Inc., Pittsburgh, PA, USA) and water (weight ratio of 25:4) was applied to the nichrome wire surface. The nichrome coil was then annealed at 80 °C for over 10 h to harden the alumina cement. The dimensions of the final tubular heater, in which the nichrome coil was embedded, were 4.7 mm in outer diameter, 3.4 mm in inner diameter, and 10 mm in length. This nichrome heater was installed in a motorized linear stage and connected to an adjustable direct current (DC) power supply (1687B, B&K Precision Co., Yorba Linda, CA, USA). In this study, the heat-scanning time (in min/cm) was calculated by dividing the overall heat-scanning time by the fiber length.

### 2.3. Characterization

The morphologies of the Ag nanomaterials were investigated by using field emission scanning electron microscopy (FE-SEM) (SIGMA, Carl Zeiss AG, Oberkochen, Germany). For cross-sectional SEM imaging, a fiber sample was cut by using a cross-section polisher (IB-19520CCP, JEOL, Ltd., Tokyo, Japan). A digital multimeter (34450A, Keysight, Santa Rosa, CA, USA) was used to measure the electrical resistance of conductive fibers. Electrical resistance per unit fiber length (Ω/cm) was calculated by dividing the measured resistance by the fiber length (6 cm). For force-sensor fibers, the force was determined from the applied force that was measured by using a force gauge (DTG-10, Digitech Co., Osaka, Japan). The capacitance of a fiber sensor unit was measured by using an LCR meter (9216A, GS Instech Co., Incheon, Korea).

### 2.4. Bending Tests

Static and cyclic bending tests were conducted to evaluate the flexibilities of the fibers. For static bending, 5 cm-long fiber samples were wrapped around circular objects with different radii. For the cyclic bending test, the 5 cm-long fiber was repeatedly subjected to bending by linearly translating one end of the fiber by 2 cm at a rate of 5 cm/s with the opposite end of the fiber fixed. This motion resulted in a bending radius of 7 mm at the fiber center. For both tests, the change in electrical resistance caused by bending was evaluated by using the digital multimeter. A strip of copper tape was attached to both ends of the fiber, and a small amount of silver paste (ELCOAT P-100, CANS Co., Tokyo, Japan) and liquid metal (gallium–indium eutectic, Sigma-Aldrich, St. Louis, MO, USA) was applied to the silver–copper interface to enhance electrical contact.

### 2.5. Fabrication of Force Sensor

A solution of 5 mM of (3-mercaptopropyl)trimethoxysilane (MPTMS, 95%, Sigma-Aldrich) in ethanol (99.8%, HPLC, Sigma-Aldrich) was used to enhance adhesion between Ag and polydimenthylsiloxane (PDMS, Sylgard 184 silicone elastomer kit, Dow, Inc., Midland, MI, USA) [30,31]. The heat-scanned fiber was immersed in this solution for 1 h, rinsed in ethanol, and dried in air. For hydrolysis and condensation, the fiber was immersed in 0.1 M hydrochloric acid (HCl, ACS reagent, 37%, Sigma Aldrich) for 1 h, followed by rinsing with DI water and then drying in air [32]. Finally, the fiber was coated with PDMS (silicone elastomer base: curing agent = 10:1) via dip coating. A fiber-type force sensor was then fabricated by stacking two conductive fibers such that they were oriented perpendicularly.

## 3. Results and Discussion

### 3.1. Heat-Scanning Fabrication of Conductive Monofilament Fibers

**Figure 1:**
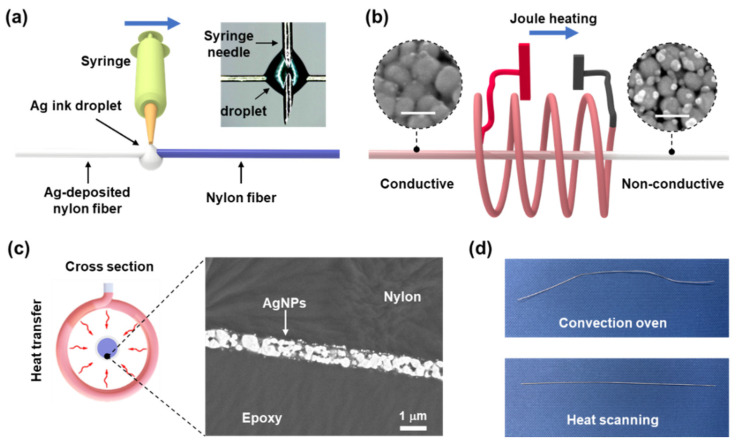
(**a**) Schematic of droplet-coating method (inset: microscopy image of the Ag ink droplet); (**b**) schematic of the heat-scanning process (inset: SEM images of sintered AgNPs (left) and as-deposited AgNPs (right), scale bar: 100 nm); (**c**) schematic of the heat transfer process (left) and the cross-sectional SEM image of an AgNP-deposited nylon fiber (right); and (**d**) digital images of fibers that were annealed by using a convection oven (150 °C) (left) and heat-scanning method (right).

For the fabrication of conductive monofilament fiber, a nylon fiber was coated with a thin layer of Ag nanomaterials by using a droplet of Ag ink [5]. Figure 1a shows a schematic of the droplet-coating method that was developed here to coat nylon fibers with AgNPs or AgNWs. The AgNP or AgNW ink was supplied via an injection needle with the needle tip holding an ink droplet (volume of 0.01 mL) that partially wetted the nylon fiber. Simultaneously, the needle was linearly translated along the longitudinal direction of the fiber, with the droplet continuously wetting the fiber surface. By this method, the fiber surface could be coated with conductive materials by using a minimal amount of Ag ink. Moreover, the amount of deposited Ag could be tuned by varying the number of sweeps, enabling control over the conductance of the resulting fiber (Figure S1).

As described in Figure 1b, the deposited Ag nanomaterials were sintered into a thin conductive layer by using the home-built nichrome heater. At sintering temperatures, the insulating polyvinylpyrrolidone (PVP) layer that covered the Ag nanomaterial was removed. The Ag-deposited nylon fiber was installed in alignment with the center axis of the nichrome heater. The application of DC power to the nichrome heater caused its temperature to rapidly increase by Joule heating. While the heat transferred from the hot nichrome heater induced the sintering (Figure 1c), a motorized linear stage translated the hot nichrome heater back and forth along the fiber at a speed of 5 cm/s. This heat-scanning motion was necessary not only to induce sintering of the Ag materials along the entire length of the fiber, but also to avoid thermal damage to the nylon fiber. Fibers prepared without this scanning motion were subjected to a steady heat flux that severely deformed the nylon fiber. Changing the direction of the heat scanning involved deceleration of the heater. Thus, the ends of the fiber were exposed to heat for a longer time than the center of the fiber, which eventually caused the fiber to fracture. For this reason, both ends of the fiber were protected with copper tape. The thickness of the sintered AgNP layer (7.2 W, 0.5 min/cm) was measured to be ~700 nm by using SEM (Figure 1c).

The heat-scanning method was effective in sintering the coated AgNP layer with minimal thermal deformation of the core nylon fiber, as compared to conventional bulk heating methods. For instance, when heated by using a convection oven for 10 min, a 10 cm-long nylon fiber coated with AgNPs was deformed even at a threshold temperature for sintering of the AgNPs (~150 °C, see Figure S2), which is higher than the glass transition temperatures of nylon (45–70 °C). While this deformed fiber exhibited a resistance of 12.9 Ω/cm, an AgNP-deposited fiber that was annealed by using the heat-scanning method (7.2 W, 0.5 min/cm) was straight and had minimal deformation (Figure 1d), and its resistance was relatively lower (6.3 Ω/cm on average).

The above findings suggest that heat scanning induces sintering at the fiber surface without excessively heating the nylon core. The mechanism for this heat transfer can be explained based on thermal radiation, conduction, and convection. While the translation speed for heat scanning mainly affects conductive and convective heat transfer, radiative heat transfer is determined by the emissivity values of the fiber and heater surfaces, as well as the temperature difference between the two surfaces. For a AgNP film prepared by coating with a dispersed AgNP solution or AgNP ink, multiple studies reported a stark decrease in the absorption of IR for sintered films. For instance, Gao et al. found a significant decrease in the IR emissivity (from 0.79 to 0.02) of a AgNP film after sintering [33], and Reenaers et al. reported a similar decrease in emissivity and increase in near-IR reflection after sintering [34]. This mechanism suggests that subjecting a AgNP film to predominantly IR radiation efficiently heats the AgNPs at the initial stage of sintering, whereas heat absorption rapidly diminishes as sintering proceeds. This self-limiting absorption of NIR in a AgNP layer can account for the sintering of the AgNPs deposited on the nylon fiber. Initially, thermal radiation from the inner surface of the tubular heater quickly induces sintering of the deposited AgNPs. However, as the nanoparticles are sintered with repeated sweeps, the sintered Ag film protects the nylon core from excessive heating by reflecting radiative heat.

**Figure 2:**
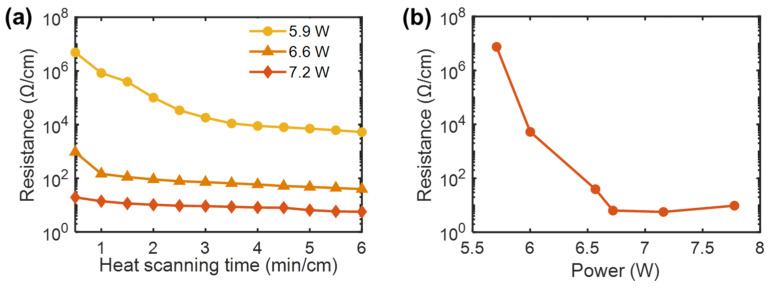
(**a**) Changes in the electrical resistances for AgNP-deposited fibers prepared by using various heat-scanning times and powers, and (**b**) electrical resistances for the AgNP-deposited fibers prepared by heat-scanning at various powers for 6 min/cm.

Sintering of the AgNPs was established by using FE-SEM, as shown in the inset of Figure 1b, and the size distribution of the sintered AgNPs was obtained (Figure S3). Energy-dispersive X-ray spectroscopy (EDX) analysis of the AgNP layer revealed that Ag occupied ~98% of the material weight (Figure S4). The electrical conductance of the coated fiber markedly increases with heat scanning because the neck structure generated by sintering improves NP-to-NP connectivity. The fiber conductance was controlled by the heat-scanning parameters. Figure 2a shows the variation of electrical resistance with heater power and heat-scanning time at a fixed scanning speed of 5 cm/s. These results indicate that the electrical resistance of the AgNP-deposited nylon fiber decreases with increasing sintering time and heat-scanning power. Figure 2b shows the variation in resistance with different heat-scanning powers for 6 min/cm of heat scanning. This reveals that the minimum electrical resistance is obtained at heating powers of ~7.2 W. Specifically, a fiber with a resistance of 5.7 Ω/cm was produced by using a heating power of 7.2 W. At this power, the temperature on the inner surface of the heater was approximately 410 °C. Fibers fractured during heat scanning when powers above 7.8 W were used, due to excessive heat input to the core nylon.

The conductance of the fibers was further improved by additionally depositing AgNWs onto the fiber surface via the same droplet-coating method but using a AgNW ink. Four types of Ag-deposited fibers were examined, including AgNP, AgNW, AgNP/AgNW, and AgNW/AgNP nylon fibers, to elucidate the effects of adding the AgNWs. Fibers with AgNP/AgNW coatings, in which a AgNP layer was deposited first followed by a AgNW layer, exhibited a bilayer structure. Reversing the order of AgNP and AgNW coatings produced AgNW/AgNP nylon fibers with the same bilayer structures. To enhance the adhesion of the Ag nanomaterials (which are covered with hydrophilic PVP) to the hydrophobic surface of nylon, an ethyl acetate/resorcinol adhesion promotor (denoted as ER) was applied to the fiber surface prior to coating with Ag nanomaterials [35]. For each type, five samples were prepared by heat scanning with the nichrome heater at a power of 7.2 W and a speed of 5 cm/s, and their resistances were measured.

**Figure 3:**
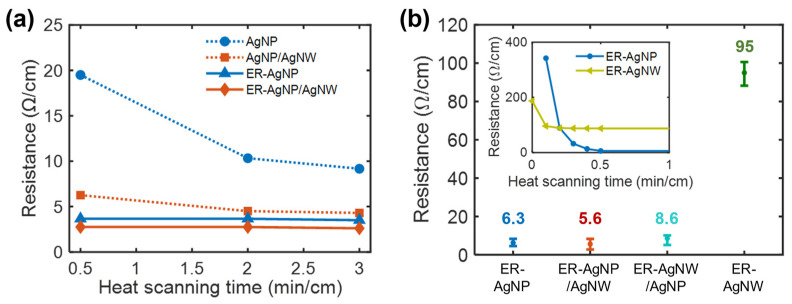
(**a**) Changes in electrical resistance with heat-scanning time (motor speed: 5 cm/s, power: 7.2 W) for the nylon fibers coated with AgNP, AgNP/AgNW, ER-AgNP, and ER-AgNP/AgNW. (**b**) Electrical resistances of the ER-AgNP, ER-AgNP/AgNW, ER-AgNW/AgNP, and ER-AgNW nylon fibers (five samples for each case) that were heat-scanned for 0.5 min/cm (inset: the initial resistance changes with heat-scanning time for ER-AgNP and ER-AgNW). The numbers inserted above the data points indicate the average resistance for each type.

**Figure 4:**
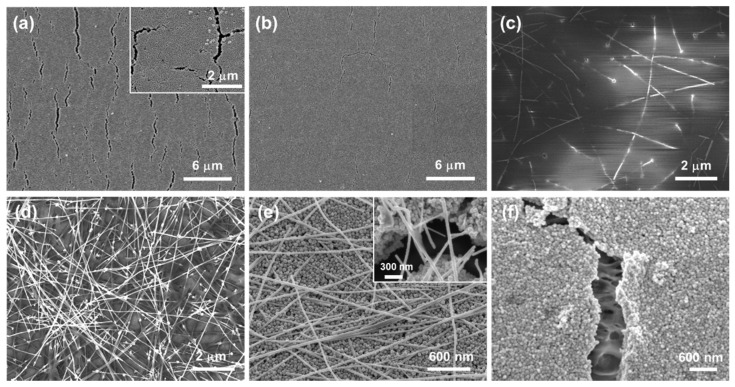
Top-view FE-SEM images that show the sintered surface of nylon fibers coated with (**a**) AgNP; (**b**) ER-AgNP; (**c**) AgNW; (**d**) ER-AgNW; (**e**) AgNP/AgNW; and (**f**) ER-AgNW/AgNP.

Figure 3a shows the changes in electrical resistance of the sample that exhibited the smallest resistance of each Ag-deposited nylon fiber, and Figure 3b compares the electrical resistances of the ER-applied fibers. Figure 4 shows representative SEM images of the fibers. For all samples, the resistance decreased with heat-scanning time. The ER treatment significantly reduced the resistance for the AgNP nylon fiber. Figure 4a,b reveal that this arose from crack generation, which was caused by the poor adhesion of AgNPs to the underlying nylon surface, that was suppressed by the ER treatment (Figure 4b). When a pristine nylon surface was directly coated with AgNWs, the density of the deposited AgNWs was too low for the AgNWs to form a conductive layer (Figure 4c). However, the ER treatment dramatically improved the adhesion of AgNWs (Figure 4d). The resistances of ER-treated fibers were markedly reduced within 0.5 min/cm of heat-scanning time (inset of Figure 3b) and specifically exhibited resistances below ~10 Ω/cm after 0.5 min/cm of heat-scanning time, except for the fiber coated with AgNWs only (~95 Ω/cm on average). The lowest resistance of 2.8 Ω/cm (0.25 Ω/sq in sheet resistance) was obtained from one of the ER-AgNP/AgNW samples. Additional heat scanning could gradually, but moderately, decrease resistance.

For ER-AgNP/AgNW nylon fibers, the addition of AgNWs to the deposited AgNP layer moderately reduced the resistance of the fiber. For instance, the resistance of the ER-AgNP/AgNW nylon fiber was 5.6 Ω/cm on average after 0.5 min/cm of heat-scanning time, which is lower than that of the ER-AgNP nylon fiber (6.3 Ω/cm). Inspection by SEM (Figure 4e) revealed that the AgNWs bridged the cracks of the supporting AgNP layer, resulting in increased conductance. A similar effect could be expected for the ER-AgNW/AgNP nylon fibers (Figure 4f), but the conductance of such fibers was lower than that of the ER-AgNP/AgNW nylon fiber. SEM micrographs (Figure S5) revealed severe cracking in the AgNP layer of ER-AgNP/AgNW nylon fiber, when compared to ER-AgNP or ER-AgNP/AgNW samples, which possibly explains the reduced conductance. This effect arose because the adhesion of the AgNP layer was hindered by the AgNWs, which were deposited prior to AgNP deposition and covered the ER-treated surface.

Comparing the resistances of ER-AgNP and ER-AgNW (Figure 3b), coating with AgNPs was more effective to obtain fibers with high conductance values. This can be ascribed to the higher coverage and loading of AgNPs per coating sweep than AgNWs. The conductance of a AgNP layer increased with the number of droplet-coating sweeps; therefore, the conductance of the ER-AgNW nylon fiber could be enhanced simply by increasing the number of coating sweeps with a AgNW ink droplet, or the loading of AgNWs (Figure S6a). For instance, the density of the deposited AgNWs increased from ~8.9 to ~15.9 μg/cm^2^ by increasing the number of coating sweeps from 16 to 42. As a result, the resistance was reduced to 17.3 Ω/cm by 0.5 min/cm of heat-scanning time (Figure S6b). However, this approach was not explored further, because increasing the number of coating sweeps leads to lengthy processing times. As a solution, we envision the installation of multiple droplet-coating spots along the fiber translation path, which could be realized in a large-scale production system.

### 3.2. Bending Performances of the Ag-Deposited Fibers

**Figure 5:**
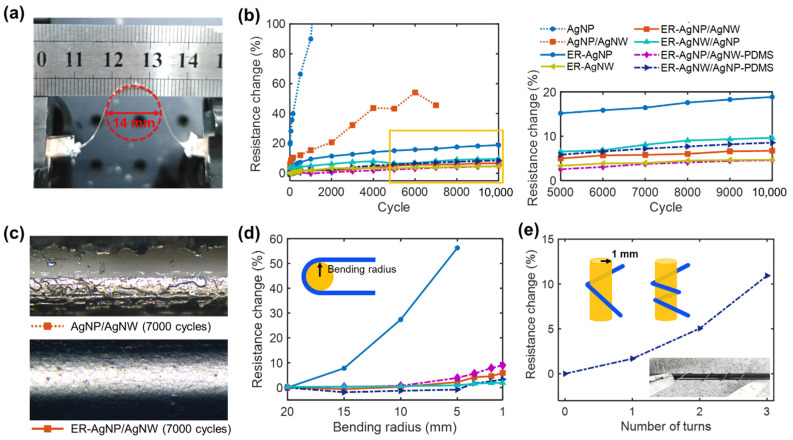
(**a**) Photograph of the cyclic bending setup with a fiber in a bent state. (**b**) The changes in electrical resistance (%) of various Ag-deposited fibers for 10,000 bending cycles (the plot on the right side corresponds to the marked area of the plot on the left side). (**c**) Optical microscopy images of AgNPs/AgNWs nylon fibers after 7000 bending cycles without and with the adhesion promoter (ER). (**d**) Electrical resistance changes of the Ag-deposited fibers with different static bending radii. (**e**) Electrical resistance changes of the ER-AgNWs/AgNPs-PDMS sample with spiral turns up to 3 times. The legend of (**b**) commonly corresponds to (**b**,**d**,**e**).

The addition of AgNWs and use of the adhesion promotors (i.e., ER treatment) not only reduced fiber resistance, but also significantly improved the flexibility of the conductive fiber. This effect was investigated by using cyclic bending tests to evaluate the flexibility of Ag-deposited fibers (5 cm long) prepared at a heater power of 7.2 W and a heat-scanning time of 0.5 min/cm. Repeated bending at a radius of ~7 mm was applied to the fibers (Figure 5a). The electrical resistance of each fiber was measured in an unbent state during the test, and the change in resistance was considered to be an indicator of its flexibility. The change in resistance was calculated by using the equation: (R − R_0_)/R_0_, where R and R_0_ are the resistance values measured after cyclic bending and the initial resistance, respectively.

Figure 5b shows the changes in resistance of various Ag-deposited fibers with bending cycles. The results reveal that the addition of AgNWs markedly suppresses changes in resistance because the flexibility of the AgNW is superior to that of sintered AgNPs. The effect of the ER treatment was more dramatic. While the AgNP and AgNP/AgNW nylon fibers prepared without ER treatment exhibited serious increases in resistance during bending tests, the changes in resistance for ER-treated Ag-deposited fibers were maintained below 20% for 10,000 cycles. For instance, the electrical resistances of ER-AgNPs, ER-AgNP/AgNW, and ER-AgNW/AgNP nylon fibers were changed by 18.8%, 6.7%, and 9.6%, respectively. Figure 5c shows optical microscopy images of the AgNP/AgNW and ER-AgNP/AgNW nylon fibers that were bent for 10,000 cycles. The images show that, without ER, the intrinsic adhesion between nylon and the Ag nanomaterials was not high enough to withstand repeated strains, resulting in delamination of the deposited Ag layer.

The enhanced flexibility for fibers added with AgNWs was pronounced at high curvatures (1/r). Figure 5d shows the results of the static bending tests, in which 180° bending was imposed on the ER-treated fibers at a range of radii, as described in the inset. The electrical resistance exponentially increased with decreasing bending radius. For the ER-AgNP nylon fiber, large resistance changes were observed at small radii. For example, the resistance change increased by over 50% at a 5 mm bending radius. By comparison, for the ER-AgNW/AgNP and ER-AgNP/AgNW nylon fibers, the changes in resistance were approximately 1.9 and 5.9%, respectively, even at a 1 mm bending radius.

The Ag-deposited fibers were coated with a protective PDMS layer (avg. thickness: ~27 μm), and their bendability was evaluated. The protective layer is necessary because the deposited Ag layer is vulnerable to abrasion, which commonly occurs in practical applications. Cyclic bending of PDMS-coated ER-AgNP/AgNW and ER-AgNW/AgNP nylon fibers (denoted as ER-AgNP/AgNW-PDMS and ER-AgNW/AgNP-PDMS, respectively) resulted in slightly reduced changes in resistance, from 6.7% to 4.6% and from 9.6% to 8.5%, respectively (Figure 5b). For static bending, the PDMS coating did not reduce the resistance, but in fact increased the resistance (Figure 5d). This possibly arises from the increased fiber diameter as a result of the PDMS coating. Figure 5e shows the change in the resistance of this fiber as it was wound multiple times around a 1 mm-radius column.

### 3.3. Application in Fiber-Type Force Sensor

**Figure 6:**
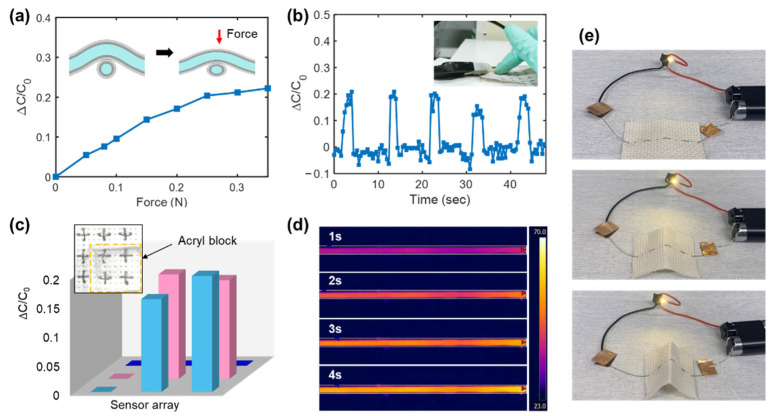
(**a**) Changes in the capacitance of a fiber sensor unit under applied force in a normal direction. The inset describes the cross section of the sensor unit. (**b**) Changes in the capacitance of the sensor unit when it was repeatedly and gently pressed with an index finger, as shown in the inset. (**c**) Changes in capacitance at each point of 3 × 3 sensor arrays when an acryl block was placed on the sensors. (**d**) Infrared thermal images of an ER-AgNP/AgNW-PDMS nylon fiber that was heated for 4 s. (**e**) Photographs of the light-emitting device (LED) powered through the conductive fiber that was subjected to bending.

Conductive fibers were examined for applications as fiber-type force sensors. Two identical conductive fibers (ER-AgNP/AgNW-PDMS) were prepared by heat scanning at a heater power of ~7.2 W for a heat-scanning time of 0.5 min/cm and stacked perpendicularly, as described in the inset of Figure 6a. The PDMS layers mediated contact between the conductive fibers in the crossed configuration. PDMS is a dielectric elastomer that is widely used as a dielectric material for soft capacitors [3]. Thus, an external force on the fibers and the resulting compressive deformation of the PDMS decreased the distance between the two Ag electrodes and yielded an increase in capacitance. Figure 6a shows the measured relationship between the capacitance and applied force in a normal direction, which agrees with this mechanism. The change in capacitance was calculated by the equation (C − C_0_)/ C_0_, where C and C_0_ indicate the capacitance with and without a load, respectively. Figure 6b shows the dynamic change of the response signal as a gentle force was repeatedly applied (by pressing on the device with a finger) to the sensor junction. Multiple force sensors (3 × 3 sensor arrays) could be installed in an embroidery fabric, as shown in the inset of Figure 6c. When a 5 mm-thick acryl block was placed on the sensor arrays, the capacitance changes for the loaded points were immediately detected (Figure 6c).

The conductive fiber could separately serve as heater filaments and flexible interconnects. Figure 6d shows a series of fiber images captured using an IR camera (FLIR A655sc, FLIR Systems, Inc., Wilsonville, OR, USA). A current of 70 mA was applied to the fiber, and it reached steady state temperature within 4 s. Figure 6e shows a series of photographs of the light-emitting device (LED) that was activated by electrical power delivered by an ER-AgNP/AgNW-PDMS nylon fiber woven in a fabric. The excellent flexibility of the fiber enabled device operation to be maintained even when the fabric was deformed.

## 4. Conclusions

In summary, Ag-deposited conductive nylon fibers were fabricated by using novel droplet-coating and heat-scanning methods. A nylon monofilament fiber was coated with a layer of Ag nanomaterial by linearly sweeping the fiber with a droplet of AgNP or AgNW ink. This droplet-coating method enabled the minimal use of Ag nanomaterials for coating. The deposited Ag layer was successfully sintered by heat-scanning the fiber with a nichrome wire-embedded tubular heater. As a result, a conductive fiber with a resistance as low as ~2.8 Ω/cm and outstanding flexibility was produced. The coating and sintering processes can be combined by translating the fiber sequentially through multiple coating droplets and tubular heaters. Moreover, these processes can be performed under ambient conditions. Thus, these methods are amenable to an in-line fabrication scheme that resembles roll-to-roll fabrication for printed electronics and that could realize large-scale production.

## Supplementary Materials

The following are available online at https://www.mdpi.com/article/10.3390/polym13091405/s1, Figure S1. Changes in electrical resistance for AgNP-deposited nylon fibers prepared by using different numbers of AgNP coating sweeps (3 min/cm, 7.2 W); Figure S2. The resistances of AgNP films (size: 0.5 cm × 2 cm) sintered at different temperatures for 10 min, which reveals a threshold temperature for sintering at ~150 °C; Figure S3. Size distribution of sintered AgNPs (7.2 W, 0.5 min/cm) on a nylon fiber; Figure S4. EDX spectrum of the deposited AgNP layer (left) and the calculated elemental composition (right); Figure S5. Optical microscopy images and SEM images for (a,c) ER-AgNP/AgNW and (b,d) ER-AgNW/AgNP; Figure S6. (a) Changes in resistance for ER-AgNW nylon fibers prepared by using different numbers of coating sweeps (0.5 min/cm, 7.2 W). (b) Changes in resistance across heat scanning time for the ER-AgNW nylon fibers prepared with 16 and 42 coating sweeps.
