# Supplementary material for: Heat Scanning for the Fabrication of Conductive Fibers
Source: Polymers (Basel). 2021 Apr 26;13(9):1405. doi: 10.3390/polym13091405 (PMC8123635; doi:10.3390/polym13091405)
Supplement: Supplementary file 1 [file polymers-13-01405-s001.zip › polymers-1196007-supplementary.pdf]

polymers

Supplementary Material

Heat Scanning for the Fabrication of Conductive Fibers

Jina Jang [1], Haoyu Zhou [2], Jungbae Lee [1], Hakgae Kim [1] and Jung Bin In [1,2,*]

[1] Soft Energy Systems and Laser Applications Laboratory, School of Mechanical Engineering, Chung-Ang University, Seoul 06974, Korea; jangjina94@cau.ac.kr (J.J.); skzzjd@cau.ac.kr (J.L.); haggai514@cau.ac.kr (H.K.)
[2] Department of Intelligent Energy and Industry, Chung-Ang University, Seoul 06974, Korea; zhouhaoyu@cau.ac.kr
* Correspondence: jbin@cau.ac.kr

Citation: Jang, J.; Zhou, H.; Lee, J.; Kim, H.; In, J.B. Heat Scanning for the Fabrication of Conductive Fibers. *Polymers* **2021**, *13*, 1405. https://doi.org/10.3390/polym13091405

Academic Editor: Victor Tcherdyntsev

Received: 8 April 2021
Accepted: 25 April 2021
Published: 26 April 2021

Publisher's Note: MDPI stays neutral with regard to jurisdictional claims in published maps and institutional affiliations.

Copyright: © 2021 by the authors. Licensee MDPI, Basel, Switzerland. This article is an open access article distributed under the terms and conditions of the Creative Commons Attribution (CC BY) license (http://creativecommons.org/licenses/by/4.0/).

Resistance change with coating sweeps

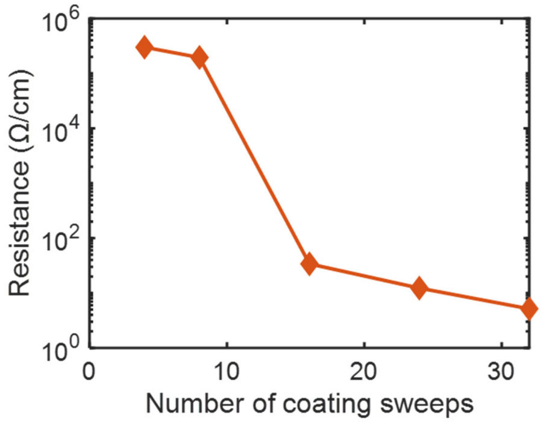

Figure S1. Changes in electrical resistance for AgNP-deposited nylon fibers prepared by using different numbers of AgNP coating sweeps (3 min/cm, 7.2 W).

Thermal damage of hotplate sintering

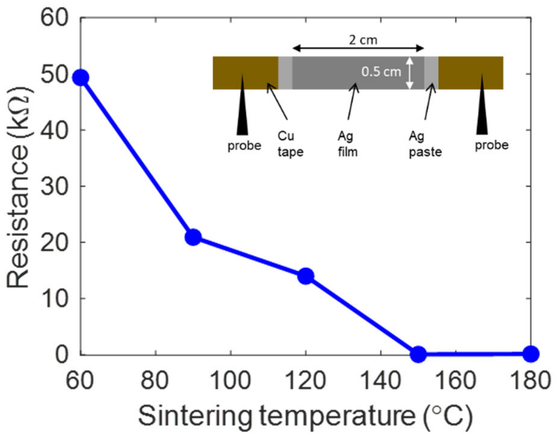

Figure S2. The resistances of AgNP films (size: 0.5 cm × 2 cm) sintered at different temperatures for 10 min, which reveals a threshold temperature for sintering at ~150 °C.

Size distribution of AgNPs

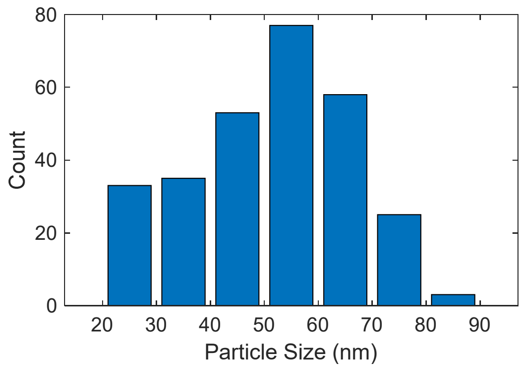

Figure S3. Size distribution of sintered AgNPs (7.2 W, 0.5 min/cm) on a nylon fiber.

Composition of deposited AgNP layer

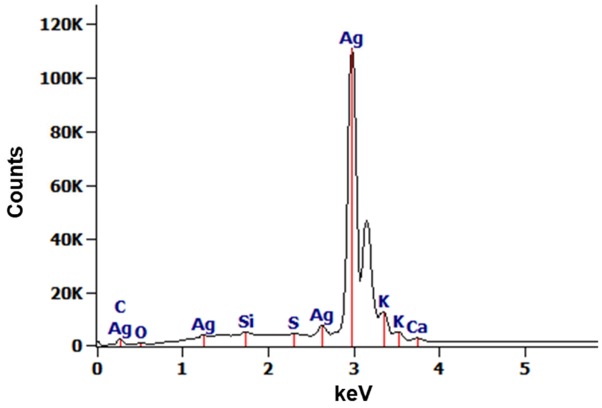

Element	Weight (%)
C	0.30
O	0.75
Ag	98.38
Si	0.29

Figure S4. EDX spectrum of the deposited AgNP layer (left) and the calculated elemental composition (right).

ER-AgNP/AgNW and ER-AgNW/AgNP

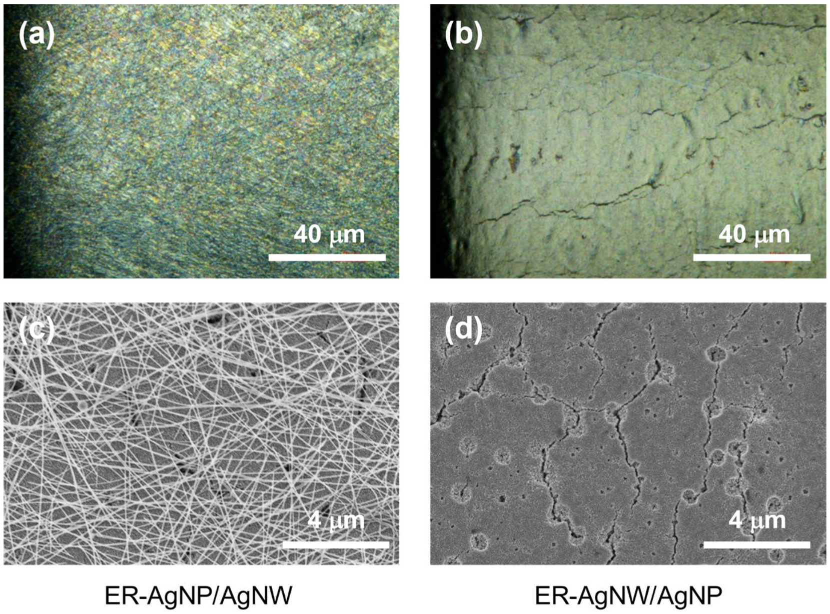

Figure S5. Optical microscopy images and SEM images for (**a, c**) ER-AgNP/AgNW and (**b, d**) ER-AgNW/AgNP.

Changes in resistance with coating sweeps for ER-AgNW fibers

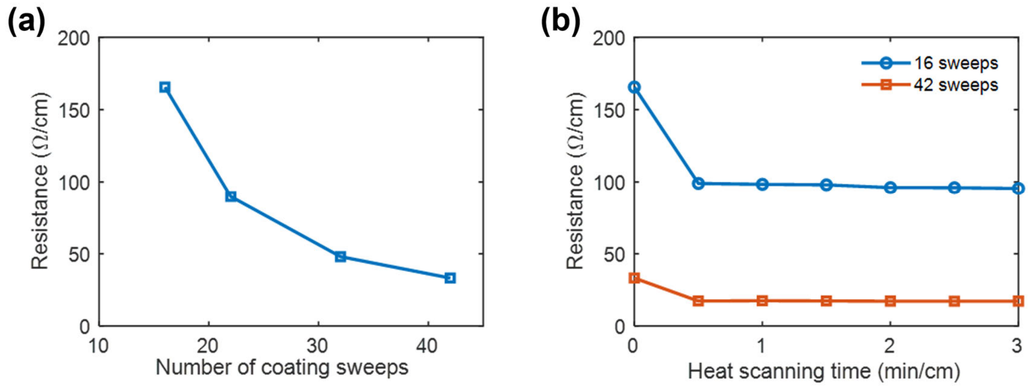

Figure S6. (**a**) Changes in resistance for ER-AgNW nylon fibers prepared by using different numbers of coating sweeps (0.5 min/cm, 7.2 W). (**b**) Changes in resistance across heat scanning time for the ER-AgNW nylon fibers prepared with 16 and 42 coating sweeps.
